# Does the chromosomal position of 35S rDNA sites influence their transcription? A survey on *Nothoscordum* species (Amaryllidaceae)

**DOI:** 10.1590/1678-4685-GMB-2018-0194

**Published:** 2020-03-06

**Authors:** Mariana Báez, Gustavo Souza, Marcelo Guerra

**Affiliations:** 1 Universidade Federal de Pernambuco, Departamento de Botânica, Laboratório de Citogenética e Evolução de Plantas, Recife, PE, Brazil.

**Keywords:** Nucleolus, ribosomal genes, preferential activation of NORs, Nothoscordum

## Abstract

35S ribosomal DNA (rDNA) sites are the regions where the ribosomal genes 18S, 5.8S and 25S, responsible for the formation of the nucleoli, are found. The fact that rDNA sites have non-random distribution on chromosomes suggests that their positions may influence their transcription. To identify if the preferentially transcribed rDNA sites occupy specific position, six species (nine cytotypes) of the genus *Nothoscordum* were analyzed using two different techniques to impregnate the nucleolar organizer regions (NORs) with silver nitrate. Both techniques strongly stained NORs, but one of them also stained the proximal region of all chromosomes, suggesting the existence of another group of argentophilic proteins in this region. In species with rDNA sites in acrocentric and metacentric chromosomes, sites located on the short arms of the acrocentric chromosomes were preferentially activated. On the other hand, in species with rDNA sites restricted to the short arms of the acrocentrics, all of them were activated, whereas in those species with sites restricted to the terminal region of metacentric chromosomes, the frequency of active sites was always lower than expected. This indicate that, at least in *Nothoscordum,* the transcription of an rDNA site is influenced by its chromosomal position, and may explain, at least partially, the strongly non-random distribution of these sites in plant and animal chromosomes.

## Introduction

Ribosomal genes play an essential role in all cells, since they form the ribosomes and participate in the transcription process of all messenger RNAs. The 18S, 5.8S and 25S ribosomal genes are assembled in a transcription unit referred to as 35S or 45S, while the ribosomal 5S gene forms an independent locus. Both 5S rDNA and 35S/45S rDNA are found repeated in tandem hundreds to thousands of times forming one or more rDNA sites per genome (Roa and Guerra, 2012). In plants, the sedimentation constant of the rDNA repeat unit is around 35S (Seitz and Seitz, 1979). Transcription of these genes, maturation of RNAs, and assembly of ribosomal subunits occur in the nucleolus (Pontvianne *et al.*, 2016). In general, the number of copies of these genes is higher than necessary for the cell, resulting that not all copies or even all rDNA sites are obligatory activated for the formation of the nucleolus, especially when there are many sites in the chromosomal complement (Brasileiro-Vidal *et al.*, 2003; Scaldaferro *et al.*, 2015).

Activation or silencing of ribosomal genes is controlled by a combination of DNA methylation, histone modifications, chromatin remodeling, and action of non-coding RNAs (Tucker *et al.*, 2010). The mechanism that controls the rRNA gene transcription seems to be based on selective silencing rather than selective activation and operates on rDNA sites instead of individual genes (Prieto and McStay, 2008; Chandrasekhara *et al.*, 2016). However, the factors that determine the preferential activation or silencing of certain sites are not well known.

In interspecific hybrids, it is common that sites of only one of the ancestors are activated while the other sites are epigenetically silenced. This type of dominance of one rDNA site over another, called amphiplasty, has been tentatively explained in different ways. One possibility is that larger sites are preferentially activated because they accumulate more promoters on the same site, thus attracting the transcription machinery more strongly, triggering the chain activation of these genes (Zurita *et al.*, 1991). However, in *Arabidopsis thaliana* it has been demonstrated that the repression of an rDNA site is tissue-specific and independent of its size (Chandrasekhara *et al.*, 2016). Other differential silencing mechanisms include gene sequence differences that affect nucleosome positioning or the base-pairing of regulatory noncoding RNAs (Preuss *et al.*, 2008; Santoro *et al.*, 2010).

During the condensation of the chromosomes in mitosis, some of the proteins involved in the transcription of the 35S rDNA sites remain attached to these sites, resulting in the undercondensation of these chromosomal regions and the formation of secondary constrictions, also called nucleolar organizer regions (NOR). Some of these proteins are argentophilic, that is, they have high affinity for silver nitrate, allowing both nucleoli and the activated NORs to be differentially stained (Tucker *et al.*, 2010). Among the argentophilic proteins present on the NOR, the most widely known are the RNA polymerase subunit I, the 50 kDa UBF (up-stream binding factor), the 135 kDa NOR protein, and SL1 (promoter selectivity factor) (McStay, 2016).

The maximum number of nucleoli per nucleus generally coincides with the maximum number of NORs detected with silver nitrate, but it may be lower than this due to the frequent occurrence of nucleoli fusions (Moscone *et al.*, 1995; Hasterok and Maluszynska, 2000). On the other hand, the maximum number of NORs does not always coincide with the number of 35S rDNA sites, since not all sites are necessarily activated in a given cell (Moscone *et al.*, 1995). Fluorescence *in situ* hybridization (FISH) allows the detection of all sites not only the active ones. In some species, there are rDNA sites never detected with silver nitrate (inactive sites) and silver-stained chromosomal regions that do not correspond to rDNA sites (cryptic sites) (Dobigny *et al.*, 2002; Caperta *et al.*, 2007; Cabrero and Camacho, 2008; Scaldaferro *et al.*, 2015). In addition, silver nitrate may also stain other proteinaceous structures, such as the synaptonemic complex, the chromosomal scaffold, the kinetochore, and the heterochromatic regions (Pathak and Hsu, 1979; Stack *et al.*, 1991; Scaldaferro *et al.*, 2015), raising doubts about the reliability of this technique as an indicator of active NORs.

The number and position of rDNA sites observed by FISH varies between species and are often restricted to a single pair of sites located on the terminal region of the short arms of the acrocentric chromosomes (Roa and Guerra, 2012; Gerbault-Seureau *et al.*, 2017). The reasons for this preferential distribution are unknown, but it is likely that this position favors massive transcription of these genes (Mohannath *et al.*, 2016) and avoids the deleterious consequences of non-homologous chromosome rearrangements promoted by rDNA sites on the proximal regions (Pedrosa-Harand *et al.*, 2006; Gerbault-Seureau *et al.*, 2017).

Among the species of angiosperms that present a wide variation in the number and position of rDNA sites are those of the genus *Nothoscordum*. Species of this genus present metacentric (M) and acrocentric (A) chromosomes combined in different karyotype formulae and apparently related to Robertsonian translocation events (Souza *et al.*, 2009, 2012). *Nothoscordum* species with 2*n* = 10 (6M + 4A) or multiples of 10, present rDNA sites generally on the short arms of the acrocentric chromosomes whereas in those with 2*n* = 8M, or multiples of 8, the sites are located mainly on the terminal region of one or both chromosome arms (Souza *et al.*, 2010, 2012, 2016). NOR analysis with silver nitrate in *Nothoscordum* is known only in *N. gracile* 2*n* = 19 (13M + 6A) (Sato *et al.*, 1981, 1988; Souza *et al.*, 2012) and in the tetraploid cytotype of *N. pulchellum* 2*n* = 20 (12M + 8A) (Roa and Guerra, 2006), both with NORs restricted to the short arms of the acrocentrics.

In this work, the activation of rDNA sites in nine *Nothoscordum* cytotypes presenting different number and position of sites was analyzed by silver nitrate impregnation, aiming to verify if there was a preferential activation of sites localized in any specific chromosome position.

## Materials and Methods

Bulbs of all species, except *N. pulchellum*, were kindly provided by Prof. Orfeo Crosa, University of the Republic of Uruguay and cultivated in the Experimental Garden of the Department of Botany of the Federal University of Pernambuco. Vouchers of these samples are deposited in the herbaria Geraldo Mariz (UPE), of the Federal University of Pernambuco, Recife, Brazil, and Bernardo Rosengurtt (MVFA) of the University of the Republic, Montevideo, Uruguay. Table 1 shows the provenance and voucher numbers of all samples analyzed.

**Table 1:** *Nothoscordum* species analyzed with their respective access number, chromosome number, karyotype formula and provenance.

Species	Access number	2*n* (karyotype formula)	Provenance
*N. felipponei* Beauverd	OC-1173	10 (6M+4A)	Minas, Lavalleja, Uruguay
	OC-1165	10 (6M+4A)	Montevideo, Uruguay
*N. gaudichaudianum* Kunth	OC-1377	8 (8M)	Rote 3, km 175, Uruguay
	OC-1328	8 (8M)	Montevideo, Uruguay
	OC-1335	16 (16M)	Montevideo, Uruguay
*N. gracile* (Ailton) Stearn	OC-14209	19 (13M+6A)	Cambará do Sul, Rio Grande do Sul, Brazil
	OC-1711	18 (14M+4A)	Maldonado, Uruguay
*N. izaguirreae* Crosa	OC-1160	24 (24M)	Rio Santa Lucia, Lavalleja, Uruguay
*N. marchesii* Crosa	OC-1153	10 (6M+4A)	Sierra de San Miguel, Rocha, Uruguay
*N. pulchellum* Kunth	MG-1704	10 (6M+4A)	Serras das Russas, Pernambuco, Brazil
		20 (12M+8A)	

### Fluorescent *in situ* hybridization (FISH)

The location of the rDNA sites by FISH was done according to the protocol described by Souza *et al.* (2012). A 6.5 kb fragment of the 35S rDNA (18S-5.8S-25S) of *Arabidopsis thaliana* clone R2 was used as probe (Wanzenböck *et al.*, 1997) labeled with digoxigenin-11-dUTP, detected with anti-digoxigenin conjugated to FITC (sheep, Roche) and amplified with anti-sheep conjugated to FITC (Rabbit, Dako). For the accessions of *N. pulchellum* 2*n* = 10, *N. gracile* 2*n* = 18 and 2*n* = 19, data previously published were used (Guerra and Felix, 2000; Souza *et al.*, 2012, 2016). The number and position of rDNA sites of *N. pulchellum* (2*n* = 10) and *N. gracile* (2*n* = 19 and 2*n* = 18) had previously been determined for the same accessions used here (Guerra and Felix, 2000; Souza *et al.*, 2010, 2012). *Nothoscordum felipponei* (2*n* = 10) had been investigated before (Souza *et al.*, 2010), but the present accessions were different from the previous one. Therefore, the accessions of *N. felipponei*, *N. gaudichaudianum* (2*n* = 8 and 2*n* = 16), *N. marchesii* (2*n* = 10), *N. pulchellum* (2*n* = 20), and *N. izaguirreae* (2*n* = 24), had the position of the rDNA sites determined here for the first time.

### Silver nitrate impregnation

Young root tips were first pretreated with 0.2% colchicine for 24 h at 10 °C. Afterwards, they were fixed and stained following one of the two different protocols used to maximize NOR detection. In the Hizume *et al.* (1980) protocol, fixation was done in ethanol:glacial acetic acid (3:1, v/v) for 2 to 24 h at room temperature and stored at -20 °C. Fixed root tips were washed in distilled water, digested in a cellulase (Onozuka) 2%-pectinase (Sigma) 20% solution, at 37 °C for 90 min, and macerated in 45% acetic acid. Coverslip was removed in liquid nitrogen and silver nitrate impregnation was made using a fresh solution of 50% silver nitrate diluted in formaldehyde. The slides were covered with a glass coverslip and incubated at 60 °C in water bath for 40-50 min. After incubation, the coverslip was removed, the slides were air dried, mounted in glycerol, and analyzed on a Leica DMB microscope. Imaging capture was done with a Leica DFC 345 FX video camera and Leica LAS AF software. Images were edited in Adobe Photoshop CS3.

In the other protocol (Vieira *et al.*, 1990), the pretreated root tips were fixed in FAA (37% formaldehyde, 50% ethanol, and glacial acetic acid, 1:18:1, v/v) solution, for 4 h at room temperature, and stored at -20 °C. The root tips were washed in distilled water, immersed in a 20% silver nitrate solution, at 60 °C for 24 h, washed in distilled water, and exposed to 1% hydroquinone diluted in 10% formaldehyde (1:1 w/v) for 10-15 min. Then, they were squashed in 45% acetic acid, mounted in glycerol, and analyzed as described above. These two staining techniques will be referred to herein as SI (slide impregnation - ethanol-acetic fixation followed by silver impregnation on the slide) and RI (root impregnation - FAA fixation followed by silver impregnation).

## Results

### Distribution of rDNA sites

**Figure 1:**
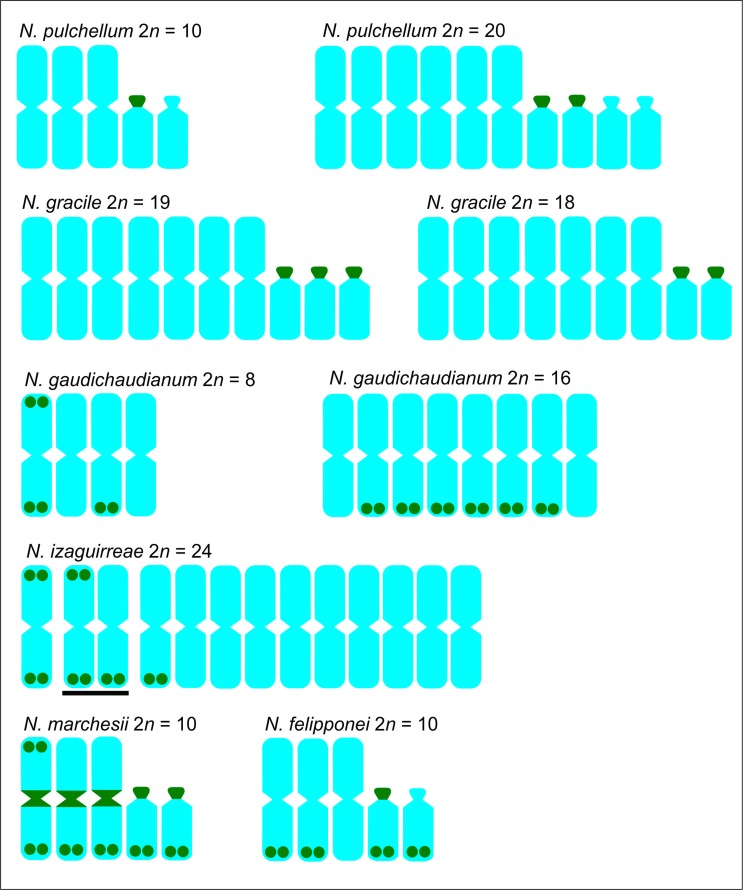
Graphic representation showing the distribution of 35S rDNA sites (green) in the analyzed species of the genus *Nothoscordum* (based on Souza *et al.*, 2012). Bar in *Nothoscordum izaguirreae* indicates heteromorphic chromosome pair.

The chromosome numbers and karyotype formulae of the analyzed accessions are presented in Table 1. The positions of the sites in these accessions are indicated in Table 2 and Figure 1. Cryptic rDNA sites, that is, NORs without corresponding rDNA sites, were never found. In relation to their chromosome position, the sites were classified as: Ap, located on the acrocentric chromosomes occupying the whole short arm; At, on the terminal region of the long arm of acrocentrics; Mt or Mp, located respectively on the terminal or proximal region of metacentrics; and Mtt when the rDNA sites were found on the terminal region of both arms of a metacentric chromosome. A total of 76 sites were found in the nine cytotypes analyzed: 30 sites on acrocentrics (22Ap + 8At) and 46 sites on metacentrics (6Mp + 24Mt + 8Mtt) (Table 2).

**Table 2:** *Nothoscordum* species analyzed with respective karyotype formula (KF), number and chromosome position of rDNA sites and NORs, and number of nucleoli per nucleus and total of nuclei analyzed. Species are organized according to the distribution of rDNA sites, as in the text.

Species	KF	Number of rDNA sites	Number of NORs	Maximum number of NORs per position				Number of nucleoli	Number of nucleoli per nucleus							Number of nuclei
			Mod - Max	Ap	At	Mp	Mt	Mod - Max	1	2	3	4	5	6	7	
**rDNA sites restricted to short arms of acrocentrics**																
*N. pulchellum*	6M+4A	2Ap	2 - 2	2	-	-	-	1 - 2	5830	1392						7222
	12M+8A	4Ap	4 - 4	4	-	-	-	2 - 4	191	265	130	27				613
*N. gracile*	13M+6A	6Ap	6 - 6	6	-	-	-	1 - 6	574	509	181	20	7	10		1301
	14M+4A	4Ap	4 - 4	4	-	-	-	1 - 4	206	158	21	11				396
**rDNA sites on metacentric chromosomes**																
*N. gaudichaudianum*	8M	2Mt+2Mtt	3 - 5	-	-	-	5	2 - 5	171	320	247	57	6			801
	16M	12Mt	2 - 5	-	-	-	5	2 - 6	28	147	107	18	7	6		313
*N. izaguirreae*	24M	2Mt+4Mtt	7 - 7	-	-	-	7	2 - 7	16	38	33	31	14	3	3	138
**rDNA sites on metacentric and acrocentric chromosomes**																
*N. felipponei*	6M+4A	4Mt+4At+2Ap	3 - 5	2	3	-	2	2 - 4	21	47	36	2				106
*N. marchesii*	6M+4A	2Mtt + 4Mt + 6Mp + 4Ap + 4At	3 - 11	4	-	4	2	2 - 5	47	141	140	76	16			420

Ap = proximal site on acrocentric short arm; At = terminal site on acrocentric long arm; Mp = proximal site on metacentric chromosome; Mt = terminal site on metacentric long arm Mtt = metacentric terminal site on long and short arms. Mod = modal number; Max = maximum number.

**Figure 2:**
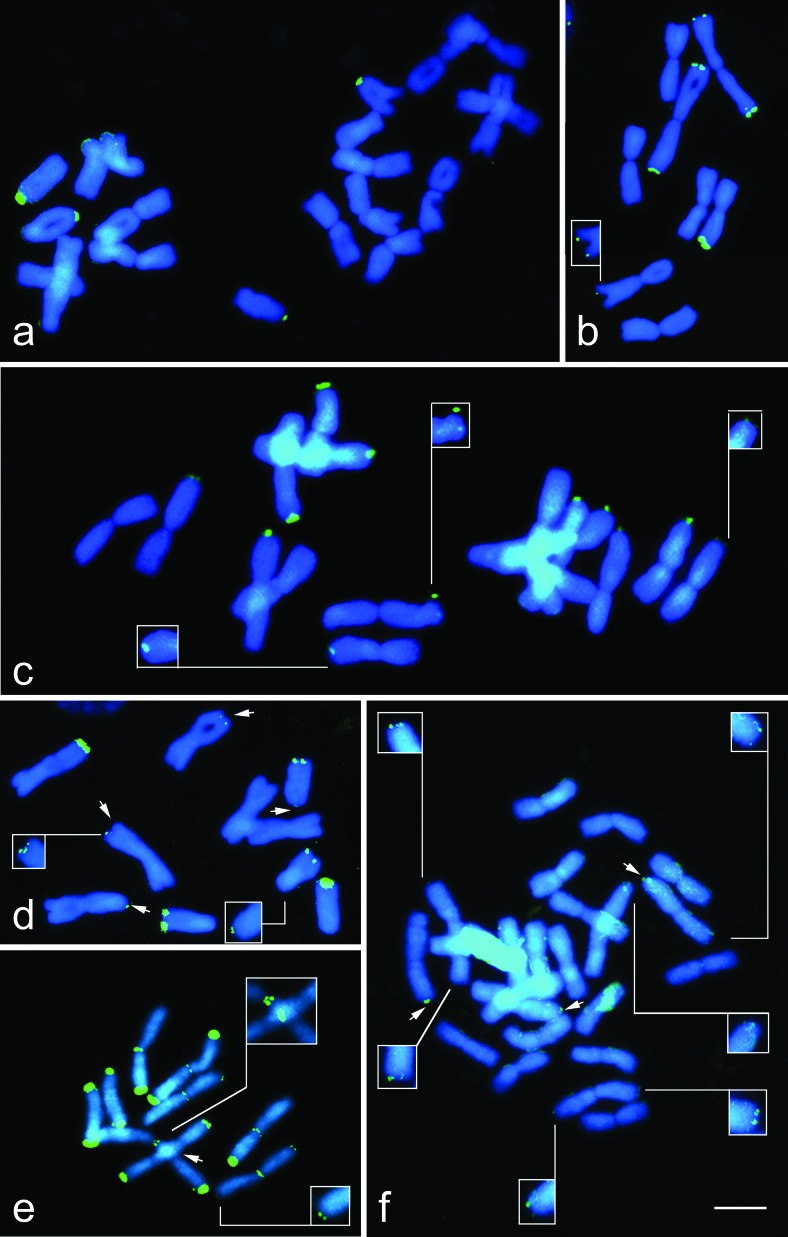
Distribution of 35S rDNA sites (green) in *Nothoscordum pulchellum* 2*n* = 20 (a); *N. gaudichaudianum* 2*n* = 8 (b); *N. gaudichaudianum* 2*n* = 16 (c); *N. felipponei* 2*n* = 10 (d); *N. marchesii* 2*n* = 10 (e); *N. izaguirreae* 2*n* = 24 (f). Observe in c an association between rDNA sites in two acrocentric chromosomes, with one of them smaller than the other. Arrows indicate small rDNA sites and insets show the sites that were not visible after merging DAPI (blue) and FITC (green) images. The scale bar corresponds to 10 μm.

For the 2*n* = 20 cytotype of *N. pulchellum* (Figure 2a), the number and position of the rDNA sites (4Ap) coincided with that expected based on the diploid cytotype (2Ap). The diploid accession (2*n* = 8M) of *N. gaudichaudianum* presented six terminal sites (2Mtt + 2Mt) (Fig. 2b), whereas the tetraploid accession (2*n* = 16M) presented 12 Mt sites, as expected, but in different positions (Figure 2c). In *N. felipponei* (2*n* = 6M + 4A) there were 10 sites (4Mt + 4At + 2Ap; Figure 2d), with very small sites observed on one pair of metacentrics and on the short arms of the acrocentrics. For *N. marchesii* (2n = 6M + 4A), 22 rDNA sites were observed (2Mtt + 4Mt + 6Mp + 4Ap + 4At) (Figure 2e). *Nothoscordum izaguirreae* (2*n* = 24M) presented nine rDNA sites (2Mt+4Mtt) (Figure 2f). This species presented weak rDNA sites in the terminal region of the chromosomes, which allowed to identify heteromorphism in a chromosomal pair (Figure 1). Figure 1 and Table 2 summarize these results.

### Silver staining of NORs and nucleoli

**Figure 3:**
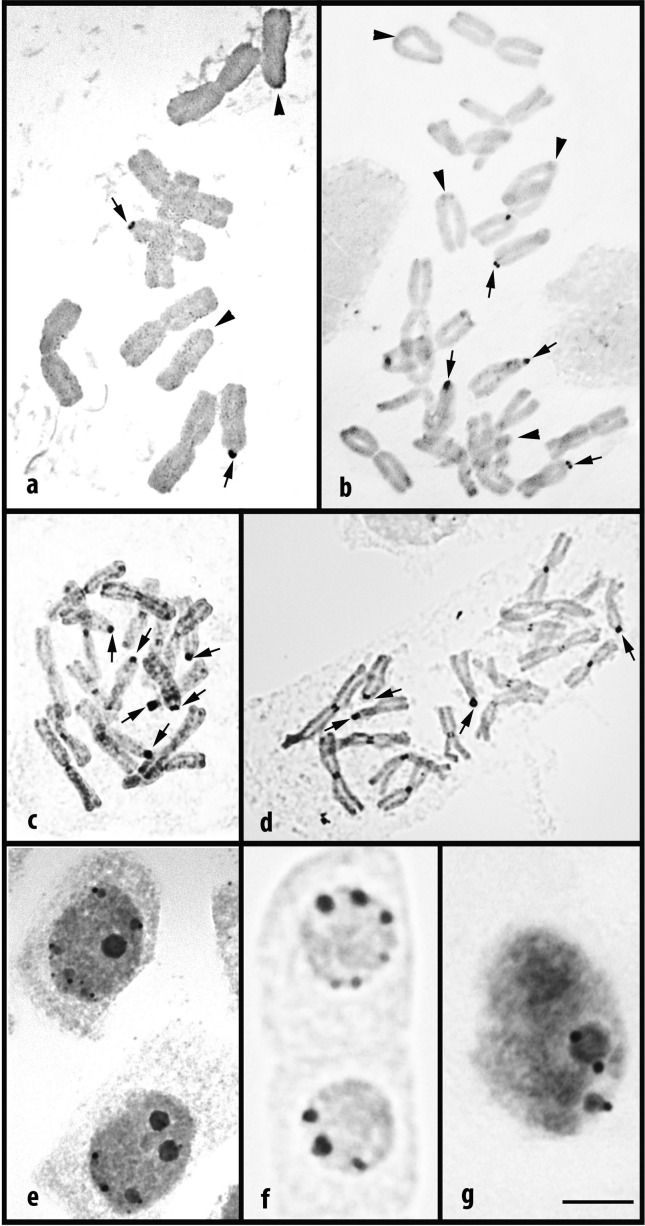
NORs and nucleoli in some species of *Nothoscordum*. Silver nitrate impregnation was done directly on slides (a, b) or on root tips (c-g). Metaphases of *N. pulchellum* 2*n* = 10 (a); *N. pulchellum* 2*n* = 20 (b); *N. gracile* 2*n* = 19 (c); *N. gracile* 2*n* = 18 (d). Nuclei of *N. marchesii* (e) and *N. felipponei* (f) with several nucleoli. Nucleus of *N. pulchellum* 2*n* = 10 (g) with two nucleoli and deeply stained nucleolus associated chromatin. Observe non-specific staining of terminal regions, in b, and pericentromeric regions, in c and d. Arrows indicate NORs, arrowheads indicate acrocentric short arms without NORs. Bar corresponds to 10 μm.

**Figure 4:**
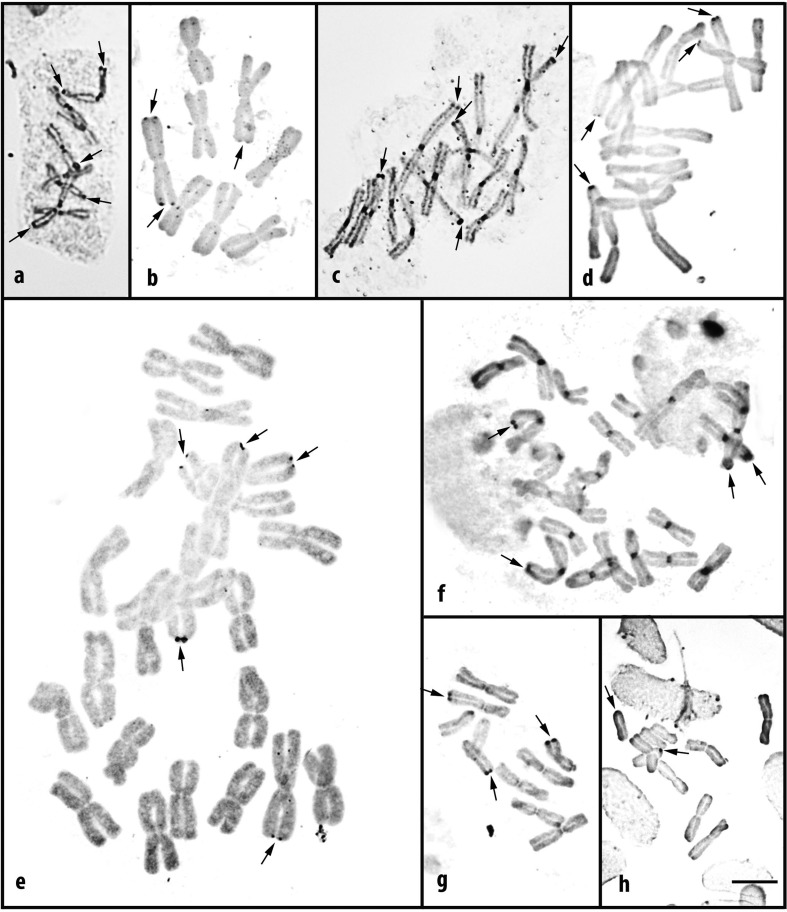
NORs (arrows) in metaphases of *Nothoscordum gaudichaudianum* 2*n* = 8 (a, b) and 2*n* = 16 (c, d); *N. izaguirreae* (e, f); *N. felipponei* (g); *N. marchesii* (h). Silver nitrate impregnation in root tips (a, c, f, g) or directly on slides (b, d, e, h). Observe in a, c, f proximal regions stained with the same intensity as NORs. Bar corresponds to 10 μm.

Both silver nitrate staining techniques used consistently revealed NORs and nucleoli. However in the RI technique the proximal regions of all chromosomes were also deeply stained (Figures 3c, d and Figures 4a, c, f). It also resulted in a greater impregnation of the chromosomes and revealed the presence of small globular structures, strongly stained, associated with the nucleoli (Figure 3g). Therefore, nucleoli number analysis was performed with RI while the number of NORs per cell was determined by SI technique, except for *N. gaudichaudianum* and *N. izaguirreae*, which have rDNA sites restricted to the terminal region of some long chromosome arms. In these cases, the proximal staining caused by RI did not interfere in the NOR detection.

### Variation in the number of nucleoli and NORs per cell

The modal number of nucleoli per nucleus varied from one in *N. pulchellum* 2*n* = 10 and *N. gracile* 2*n* = 19 and 2*n* = 18, to two in the other species (Table 2). The modal number of NORs for each accession was always greater than the modal number of nucleoli, due to a tendency to nucleoli fusion (Table 2). In general, species with many rDNA sites (9 to 22) had a lower percentage of activated sites, whereas in accessions with few rDNA sites (2 to 6), the maximum number of NORs coincided with the total number of sites, except for *N. gaudichaudianum* 2*n* = 8, with six sites and, at most, five NORs. On the other hand, in accessions with 10 to 22 sites the maximum number of NORs was always smaller than the number of rDNA sites (Table 2). However, the percentage of cells expressing the maximum number of NORs ranged from 37% to 83%, except for *N. pulchellum* 2x and 4x, which had all sites expressed in almost 100% of the cells.

### Distribution of NORs in species with rDNA sites restricted to short arms of acrocentrics

In *N. pulchellum*, only one pair of NORs in the diploid cytotype (Figure 3a) and two pairs in the tetraploid (Figure 3b) were observed, confirming the absence of the 35S rDNA sites in the second acrocentric pairs of the monoploid complement. Only two nucleoli were found, at most, in more than 7,000 nuclei of the diploid cytotype (Table 2). In *N. gracile* with 2*n* = 19 (13M + 6A) and 2*n* = 18 (14M + 4A) the modal number and the maximum number of NORs corresponded to the maximum number of rDNA sites (Table 2 and Table S1). There was no evidence of silver staining on the proximal region of the metacentrics, indicating that the fusion of acrocentrics in these accessions must have occurred on the centromere region, excluding the rDNA sites on the short arms of both acrocentrics.

### Distribution of NORs in species with rDNA sites on metacentric chromosomes

In the three accessions that presented karyotype formed only by metacentrics, the number of NORs was always smaller than expected (Table 2). In *N. gaudichaudianum* the diploid (2*n* = 8M, six rDNA sites) and tetraploid (2*n* = 16M, 12 rDNA sites) cytotypes presented, at most, five NORs per cell (Table 2, Figures 4a-d). In *N. izaguirreae* 2*n* = 24M, with nine rDNA sites, a maximum of seven NORs was found (Table 2, Figures 4e, f).

In accessions that display Mtt chromosomes, we observed that the expression of one rDNA site did not interfere with the expression of the other site located on the same chromosome. In the diploid access of *N. gaudichaudianum*, the only pair of Mtt chromosomes showed at least one of these chromosomes with the two NORs actives in 55 of the 62 cells analyzed, of which 29 corresponded to the simultaneous activation of the four sites (Table S2 and Figures 4a, b). *Nothoscordum izaguirreae*, with two pairs of Mtt chromosomes, showed a chromosome with two NORs in six of the ten cells analyzed (Table S2).

### Distribution of NORs in species with rDNA sites on metacentric and acrocentric chromosomes

In *N. felipponei* (4Mt + 4At + 2Ap), Ap sites, although very small, were active in 19 of the 58 cells analyzed (33%). The most frequently activated were At and Mt sites, with at least one active site in 93% and 74% of the cells, respectively (Figure 4g, Table S3). *Nothoscordum marchesii* had the highest number of rDNA sites (22) and NORs (11) per cell. In this accession, all cells presented Ap NORs and none of them presented At NORs. Mt and Ap sites were active in 33% and 17% of the cells, respectively (Figure 4h, Table S3).

## Discussion

### The RI technique is not specific for NORs in *Nothoscordum* species

Proximal region labeling with silver nitrate not related to NORs was observed in all *Nothoscordum* species analyzed here with the RI technique, possibly impregnating centromeric heterochromatin proteins and kinetocore proteins, as reported in other plant species (Stack *et al.*, 1991; Hizume *et al.*, 1992; Carvalho *et al.*, 2010). However, the interstitial heterochromatic bands of the acrocentric long arms of *N. gracile* (Sato, 1988; Souza *et al.*, 2012) were not impregnated with silver nitrate, suggesting that this technique does not recognize all heterochromatin types. Silver impregnation of chromosomal regions not related to NORs has been attributed to the presence of other proteins rich in acidic residues (Dobigny *et al.*, 2002), or to the occurrence of RNA polymerase I transcription factor UBF integrated at ectopic chromosome sites (Wright *et al.*, 2006). In *Nothoscordum*, the argentophilic proteins of the pericentromeric region should be different from those present in the NORs, since the first ones were not detected with SI impregnation. Noteworthy, chromosomes of *Thinopyrum ponticum* stained with the RI technique did not show proximal labeling (Brasileiro-Vidal *et al.*, 2003), suggesting that the occurrence of these proteins may be restricted to some plant groups.

### Species with many rDNA sites may include some silenced sites

In *Nothoscordum* species with 10 or more rDNA sites, the maximum number of NORs and nucleoli was always lower than the number of rDNA sites. In *N. felipponei*, with 12 rDNA sites, none of the four Mp sites were associated to NORs in the 58 cells analyzed. This suggests that the Mp sites of this species are temporarily or permanently silenced or are active only in other tissues or stages of development (Hasterok and Maluszynska, 2000; Preuss and Pikaard, 2007; Chandrasekhara *et al.*, 2016). Although a high number of rDNA sites may contain more rDNA copies than necessary, species with multiple sites may have all sites simultaneously activated, as the 17 rDNA sites in *Thinopyrum* (Brasilero-Vidal *et al.*, 2003) or the 16 sites in *Zoellnerallium andinum*, a genus proximally related to *Nothoscordum* (Souza *et al.*, 2016).

### rDNA sites located on the short arms of the acrocentric chromosomes were preferentially activated

In *Nothoscordum* species Ap sites were preferentially activated, as indicated by the following observations: 1) only cytotypes having exclusively Ap sites reached the expected maximum number of nucleoli and silver stained NORs; 2) in *N. marchesii*, with Ap, Mt, At and Mp sites, there was at least one Ap site active in all analyzed cells; 3) in general, the frequency of non-activated Mt, At or Mp sites in karyotypes having Ap sites was higher than in those without Ap sites, suggesting that in the presence of Ap sites the remaining ones were less frequently activated. Since non-activated sites are susceptible to progressive elimination (Kovarik *et al.*, 2008), it is possible that *Nothoscordum* species with Ap plus Mt, At or Mp sites tend to preserve only Ap sites. The preferential elimination of non-Ap sites could explain the low frequency of Mt, At or Mp sites in species of *Nothoscordum* and closely related genera with acrocentric chromosomes (Souza *et al.*, 2016 and references herein).

### Different factors may contribute to the preferential location of the 35S rDNA sites on the short arms of the acrocentrics

The distribution of the 35S rDNA sites along the chromosomes is clearly non-random, being located on the terminal region of the chromosome arms in 70% of the angiosperm species (Roa and Guerra, 2012). The reason for this preferential distribution is still not clear. It seems that the terminal position has at least one advantage: it is the only chromosomal position that allows the occurrence of non-homologous recombination, necessary for interlocus homogenization of rDNA repeats, without disturb the linkage of other genes (Pedrosa-Harand *et al.*, 2006). Moreover, nucleolus-related telomeric and subtelomeric sequences contribute to the regulation of rDNA gene transcription by RNA polymerase II and to the stability of the nucleolus (Pontvianne *et al.*, 2016; Picart and Pontvianne, 2017). However, if the terminal position of 35S rDNA sites is condition that is positively selected, why Ap sites, which are at the same time proximally and terminally located, are more common and more frequently activated in *Nothoscordum* than in other terminal sites?

The origin of Ap sites has been associated to different chromosomal rearrangements, such as centric fission, pericentric inversion, translocation and centromeric repositioning (Schubert and Lysak, 2011; Souza *et al.*, 2016; Chiatante *et al.*, 2017). Once established in the Ap position, 35S rDNA sites may take their advantage from their neighborhood, sandwiched between the pericentromeric and telomeric/subtelomeric heterochromatins, which would protect the protein coding genes close to NOR against an eventual propagation of the intense transcriptional activity of rDNA genes (McStay, 2016). However, several species of *Nothoscordum* and other genera display NORs on the long arms, without evidences of heterochromatin on the proximal side, suggesting that other factors may also contribute to this preferential position. Therefore, the preferential location of 35S rDNA sites on short arm of acrocentric chromosomes seems to be determined by random chromosomal rearragements whereas its preferential activation may result in an effective functional advantage, ensuring a higher stability and contributing to its higher frequency in eukaryotic chromosomes.

## Supplementary material

The following online material is available for this article:

Table S1 Number of NORs in species with rDNA sites only on acrocentric short arms

Table S2 Patterns of NORs in species with rDNA sites only in metacentric

Table S3 Patterns of NORs in species with rDNA sites in acrocentric and metacentric
