# Supplementary material for: Does the chromosomal position of 35S rDNA sites influence their transcription? A survey on Nothoscordum species (Amaryllidaceae)
Source: Genet Mol Biol. 2020 Mar 6;43(1):e20180194. doi: 10.1590/1678-4685-GMB-2018-0194 (PMC7197985; doi:10.1590/1678-4685-GMB-2018-0194)
Supplement: Supplementary file 1 [file 1415-4757-GMB-43-1-e20180194-s001.pdf]

Genetics and Molecular Biology 43, 1, (2020)

Supplementary Material to "Does the chromosomal position of 35S rDNA sites influence their transcription? A survey on Nothoscordum species (Amaryllidaceae)"

Table S1 - Number of NORs per cell in species with rDNA sites only on short arms of acrocentric chromosomes (Ap).

Species (rDNA sites)	N	Number of cells with 1 to 6 NORs					
		1	2	3	4	5	6
N. pulchellum (2Ap)	81		81				
N. pulchellum (4Ap)	67				67		
N. gracile (4Ap)	17		4	6	7		
N. gracile (6Ap)	33	1	4		11	1	16

N = number of cells analyzed
