# Supplementary material for: Does the chromosomal position of 35S rDNA sites influence their transcription? A survey on Nothoscordum species (Amaryllidaceae)
Source: Genet Mol Biol. 2020 Mar 6;43(1):e20180194. doi: 10.1590/1678-4685-GMB-2018-0194 (PMC7197985; doi:10.1590/1678-4685-GMB-2018-0194)
Supplement: Supplementary file 2 [file 1415-4757-GMB-43-1-e20180194-s002.pdf]

Genetics and Molecular Biology 43,1, (2020)

Supplementary Material to "Does the chromosomal position of 35S rDNA sites influence their transcription? A survey on Nothoscordum species (Amaryllidaceae)"

Table S2 - Patterns of active NORs in species with rDNA sites only in metacentric chromosomes. Chromosomes displaying a single terminal active NOR (t) may bear only one rDNA site (Mt) or two rDNA sites (Mtt) whereas those bearing active NORs in both termini (tt) are supposed to be Mtt. The total number of NORs per cell is shown in parentheses.

Species (rDNA sites)	N	Number of cells with different NOR pattern											
		1 t (1)	2 t (2)	1 tt (2)	3 t (3)	1 t + 1 tt (3)	4 t (4)	2 tt (4)	2 t + 1 tt (4)	5 t (5)	1 t + 2 tt (5)	6 t (6)	5 t +1 tt (7)
N. gaudichaudianum (2Mt + 2Mtt)	62	4	2	3		23	1	17	8		4		
N. gaudichaudianum (12Mt)	62		39		7		14			2			
N. izaguirreae (3Mt + 3Mtt)	10											4	6

Mt = metacentric chromosome with terminal site; Mtt = metacentric chromosome with terminal site in both arms.
