# Supplementary material for: Does the chromosomal position of 35S rDNA sites influence their transcription? A survey on Nothoscordum species (Amaryllidaceae)
Source: Genet Mol Biol. 2020 Mar 6;43(1):e20180194. doi: 10.1590/1678-4685-GMB-2018-0194 (PMC7197985; doi:10.1590/1678-4685-GMB-2018-0194)
Supplement: Supplementary file 3 [file 1415-4757-GMB-43-1-e20180194-s003.pdf]

Genetics and Molecular Biology 43,1 (2020)

Supplementary Material to "Does the chromosomal position of 35S rDNA sites influence their transcription? A survey on Nothoscordum species (Amaryllidaceae)"

Table S3 - Patterns of active NORs in species with rDNA sites in acrocentric (Ap, At, Apt) and/or metacentric (Mp, Mt, Mpt, Mtt) chromosomes. Acrocentric chromosomes with a proximal or a terminal active NOR were indicated as ap or at, respectively, and metacentrics with a single proximal or a single terminal NOR were described as mp and mt, respectively. Only a few chromosomes with a proximal and a terminal active NORs (apt or mpt) were found and no chromosome with NOR in both termini were observed. Number of NORs per cell is shown in parentheses.

Species (rDNA sites)	N	Number of cells with different NOR pattern										
N. felipponei (4Mt + 4At + 2Ap)		2 at (2)	2 ap (2)	1 at + 1 mt (2)	2 at + 1 ap (3)	2 at + 1 mt (3)	2 ap + 1 mt (3)	1 at + 1 ap + 1 mt (3)	2 t + 2 mt (4)	3 at + 1 mt (4)	2 apt + 1 ap (5)	2 apt + 1 mt (5)
	58	7	3	7	3	20	1	9	3	2	2	1
N. marchesii (2Mtt + 4Mt + 6Mp + 4Ap + 4At)		2 ap (2)	3 ap (3)	2 ap + 1 mt (3)	2 ap + 2 mp (4)	2 ap +1 mt + 4 mp (7)	4 ap + 1 mt + 2 mp (7)	4 ap + 1 mpt + 3 mp (9)	4 ap + 1 mt + 4 mp (9)	3 ap +2 mpt + 4 mp (11)	4 ap + 4 mp (8)	
	58	20	16	12	2	1	1	1	2	2	1	

Ap = acrocentric chromosome with proximal site; At = acrocentric chromosome with terminal site on the long arm; Apt = acrocentric chromosome with one proximal and one terminal sites; Mp = metacentric chromosome with proximal site; Mt = metacentric chromosome with terminal site; Mpt = metacentric chromosome with one proximal and one terminal sites; Mtt = metacentric chromosome with terminal site in both arm
